# The influence of first wave of COVID-19 outbreak on routine healthcare services, Liberia, August 2020: a mixed study approach

**DOI:** 10.1186/s12913-022-08074-3

**Published:** 2022-05-21

**Authors:** Obafemi J. Babalola, Himiede W. Sesay, Lily S. Blebo, Faith K. Whesseh, Chukwuma D. Umeokonkwo, Peter A. Adewuyi, Maame Amo-Addae

**Affiliations:** 1 African Field Epidemiology Network (AFENET), Liberia Office, 22 Russel Ave., 21 Street, Sinkor, Central Monrovia, Montserrado County Liberia; 2 Liberia Field Epidemiology Training Program, Central Monrovia, Montserrado County Liberia

**Keywords:** Coronavirus, COVID-19, Delivery of health care, Immunization, Outpatient, Qualitative study, Liberia

## Abstract

**Background:**

The COVID-19 pandemic left countries to rapidly implement diverse and stringent public health measures without recourse to mitigate its effect on the sustenance of routine healthcare services. This study described routine health service disruption and restoration strategies at 6 months into the epidemic in Liberia.

**Methods:**

Liberia, with 15 counties, has 839 health facilities, with one-third in Montserrado County. A cross-sectional study using a mixed approach - quantitative and qualitative research with concurrent triangulation was conducted using a structured guide for group discussions among key health workers at 42 secondary and most patronized health facilities in 14 counties and 7 Montserrado districts. Additionally, routine health data between January and June 2019 and 2020 were extracted from the source documents to the electronic checklist. We performed a descriptive analysis of quantitative data and plotted the line graph of the relative percentage change. Transcribed audio recording notes were synthesized using ATLAS ti for content analysis to identify the themes and subthemes in line with the study objectives and excerpts presented in the results.

**Results:**

Liberia declared COVID-19 outbreak on March 16, 2020. From conducted interviews at 41 health facilities, 80% reported disruption in routine health services. From January to June 2020, scheduled routine immunization outreaches conducted decreased by 47%. Using a relative percentage change, outpatient attendance decreased by 32% in May, inpatient admission by 30% in April, malaria diagnosis and treatment by 40% in April, and routine antenatal obstetric care by 28% in April. The fear of contacting COVID-19 infection, redeployment of healthcare workers to COVID-19 response, restriction of movement due to lockdown, inadequate or lack of PPE for healthcare workers, lack of drugs and vaccine supplies for clients, and partial closure of routine healthcare services were common perceived reasons for disruptions. Massive community health education and strict compliance with COVID-19 nonpharmacological measures were some of the health facility recovery strategies.

**Conclusions:**

The COVID-19 outbreak in Liberia caused a disruption in routine healthcare services, and strategies to redirect the restoration of routine healthcare services were implemented. During epidemics or global health emergencies, countries should sustain routine health services and plan for them.

## Background

The novel coronavirus SARS-CoV-2 has caused a severe pandemic, affecting all facets of life [1]. According to the UNICEF Executive Director Henrietta Fore, “The COVID-19 pandemic continues to pose serious challenges to global health beyond the impact of the disease itself” [2]. Globally, as of September 18, 2020, the COVID-19 pandemic, with 31 million cases in 213 countries and territories, has overstretched the health system, resulting in a significant disruption in the utilization of routine healthcare services globally [3], but low-income countries have been more affected [4]. In Liberia, 1331 cases including 82 deaths reported as of September 18, 2020.

The declaration of COVID-19 as a global pandemic by the World Health Organization left countries to rapidly implement diverse stringent measures to combat the transmission of the disease without recourse to mitigate its indirect effect on routine healthcare services [5]. In 2020, many months after the outbreak, substantial disruptions in healthcare utilization were reported by 90% of the countries that participated in the World Health Organization’s second round of the “pulse survey” [2].. On average, half of the essential or routine health services in 2020 were disrupted with little or no change in the first quarter of 2021 [2]. Additionally, evidence from a systematic review shows that routine healthcare services were consistently disrupted with a reduction in the early phase of the pandemic period [6].

The causes of disruption in routine healthcare services vary as the outbreak progresses. At the beginning of the COVID-19 outbreak, reasons for disruption were due to emergency reorganization in the health sector to promptly respond to the outbreak [7]. However, routine healthcare services became more disrupted as the initial sporadic COVID-19 case identification became continuous with prolonged community transmission [4]. Other causes of disruption were the introduction of lockdown or restriction of movement by the government, outright closures of health facilities in cases where there was COVID-19 infection among healthcare workers, and non-availability of essential medicines, diagnostics, and personal protective equipment (PPE) needed for safe and effective healthcare delivery. The global lockdown or restriction of the movement of goods affects the global supply chain system and may be associated with routine healthcare service disruption [2].

Liberia recorded its first case of COVID-19 on March 14 and declared an outbreak on March 16, 2020 [8] and as at. It instituted measures to combat the transmission. The country’s health system infrastructure was recently disrupted during the Ebola virus disease (EVD) outbreak in 2014 [9, 10], and efforts to rebuild it were ongoing. With the lessons learned from the previous EVD experience, several measures were implemented to stop the transmission of COVID-19. This included redeployment of healthcare workers from their primary assignment to the COVID-19 outbreak response, which further created gaps in routine health services and sustained them during the outbreak [11].

Following the global evidence of COVID-19 disruption of routine healthcare services and its tendency to erode public health gains of many years, the World Health Organization (WHO) and its partners advised countries to adapt strategies to adequately respond to the routine healthcare service disruption related to the COVID-19 outbreak [2] to maintain the utilization of routine healthcare services such as malaria diagnosis and treatment, antenatal care, immunization services, cancer treatment, surgeries, reproductive health, HIV/AIDS, noncommunicable diseases, and many others [12, 13]. Therefore, in response to the WHO’s call, this assessment was rapidly conducted to describe the effect of the COVID-19 outbreak on the disruption of common routine health services, such as outpatient visits, inpatient admissions, antiretroviral therapy, malaria diagnosis and treatment, antenatal clinic attendance, routine immunization services, and reasons for disruption, and to describe recovery strategies being implemented by the health facilities to initiate the restoration of routine healthcare services in Liberia.

## Methods

### Study setting

This survey was conducted in Liberia, a West African country with an estimated population of 5.1 million people [14] and subdivided into 15 counties, 93 health districts, and 839 health facilities. Montserrado County, which is the seat of the Liberian government, is the most populous and accommodates one-third of the country’s population. It has seven districts that equate to the other 14 counties in administration.

### Study design

A cross-sectional study using a mixed approach of qualitative design and using a checklist to extract health records of routine health services for the year 2019 and 2020.

### Selection of health facility

Liberia was divided into 15 counties, but Montserrado County was the largest. It has 7 districts with administrative functions similar to those of the other 14 counties. For this study, the 14 counties and 7 districts in Montserrado were classified as sampling units, totaling 21. At each unit, public and nonpublic secondary health facilities that run all the routine healthcare services selected for analysis, mostly patronized by the people, i.e., > 1000 patients per month, designated referral centers, have a designated isolation site for persons with infectious diseases such as EVD, Lassa fever and COVID-19 before transfer to the treatment unit, were selected into the sampling frame and stratified into public and nonpublic health facilities. Thereafter, one public and one nonpublic health facility were randomly selected per county or district, and 42 health facilities were selected for the study.

For focus group discussions at the health facilities, based on the role of the healthcare workers within the selected health facilities, the Officer in charge (OIC) of the health facility, Health Facility Surveillance Focal Person (HFSFP), Vaccinator/Child Survival Focal Person, Ward Supervisors, Nursing Directors, Screeners or Triage Officers, and Record Officer or Registrars were invited for focus group discussion. However, the health workers responsible for routine health services selected for the assessment supported the data collectors to extract records from the ledgers using the checklist.

### Data collection tool

A structured focus group discussion guide was developed. It had seven questions, including “What can you say by comparing the current state of routine healthcare services in this health facilities to pre-COVID-19 era?”, “How has COVID-19 outbreak affected the delivery of routine healthcare services in this health facility?”, “What impact does COVID-19 outbreak have on client’s access to routine healthcare services?”, “What do you think changed your clients’ health-seeking behavior at this health facility?”, “What are other reasons that caused the changes that you noticed in routine healthcare service?”, “What is your health facility doing to restore to “normal” routine healthcare service delivery?”, and “Since the implementation of these changes, have you noticed any change?”. For the quantitative aspect, a checklist was developed with the Kobo Collect toolbox and indicated the; type of routine health services been assessed at the health facility, e.g., outpatient, inpatient, antenatal, routine immunization service, malaria diagnosis and treatment; and HIV/AIDS and antiretroviral therapy, the checklist was used to extract the number of clients or patients seen at various routine health service units from January to June 2020 and similar periods in 2019.

### Data collection technique

For the group discussions, 6 to 10 persons were purposefully selected from the eligible participants at the selected health facility for a scheduled time. The trained data collectors were the graduates and trainees of the Liberia Field Epidemiology Training Program (LFETP), supervised by the faculty. A moderator assisted by a timekeeper or recorder conducted the interview in English language, which was audio recorded. The notes taken during the interview were expanded by transcribing the audio recording. The transcribed notes were revalidated by the supervisor after listening to the audio recording. Furthermore, all recordings and transcribed notes were reviewed by an independent reviewer for correctness before analysis. For quantitative data, routine health records surveillance data were reviewed, and data were extracted from January to June of year 2019 and 2020 from the Diagnosis and Treatment Ledgers at the outpatient departments, Admission and Discharge Registers for inpatients, EPI ledger, antenatal logbook, and HIV treatment registers. The quantitative data were extracted from the health facilities ledgers using an electronic data collection tool, i.e., the Kobo Collect toolbox, installed on an Android phone.

### Data analysis

For qualitative data analysis, revised transcripts of the audio recordings were uploaded into Computer Assisted Qualitative Data Analysis Software, ATLAS.ti, for data management and analysis based on study objectives. A codebook was also developed and validated by an independent analyst. Thereafter, the audio recording transcripts were critically analyzed for codes, which was organized hierarchically into sub-themes and finally into three main themes grouped under the study objective’s themes. The themes were health service disruption defined as a change in number of clients receiving the health service, closure of service, and alteration of service provision. Other themes are causes of disruption and recovery strategy by the health facilities. The interpretation of results was presented with excerpts or quotes from the data. However, for the quantitative data, a relative percentage change was calculated. The relative percentage change in service delivery was defined as the difference of the initial from the latest value divided by the baseline value, using the formulas:

$$\mathrm{Percentage}\ \mathrm{Change}\ \mathrm{in}\ \mathrm{Service}\ \mathrm{delivery}=\frac{\left({\mathrm{y}}_{2020}-{\mathrm{y}}_{2019}\right)}{{\mathrm{y}}_{2019}\ }\ \mathrm{X}\ \frac{100}{1}$$

Where:

y_2020_ = number of clients served in a particular month in 2020.

y_2019_= number of clients served in the same month in 2019.

The results were plotted in deviation bar charts for each of the services over the six-month period.

### Ethical considerations

Prior to the field exercise, approval was sought from the Ministry of Health and the County Health Teams. As a rapid study to assess disruptions to routine health services while responding to the COVID-19 outbreak, approval from approval from the Ministry of Health Institutional Review Board through the Liberia COVID-19 Incidence Management System was waived with reference letter no: 058. At the health facilities, data collectors met with the health facility administrator to seek approval for data collection after informing them of the purpose of the exercise and written informed consent obtained from the participants. To maintain participant confidentiality, a code was assigned to each participant and health facility that was used during the interview and in data analysis. Data collectors and participants were issued face masks, face shields and hand sanitizers, and social distance was maintained to provide protection from COVID-19.

## Results

One of the 42 health facilities selected for the study declined participation. Most of the health facilities (73.2%, 30/41) were in urban settings. Table 1 shows the characteristics of the FGD respondents and the themes and sub-themes identified during the interview was in Table 2. Compared to January to June 2019, the COVID-19 outbreak during the same period in 2020 caused a decrease in facility attendance or visits by patients in 85.4% (35/41) of the 41 health facilities sampled. In addition, 80.0% (28/35) of these health facilities agreed that there was a disruption of routine healthcare services and a decrease in their financial income and routine surveillance data reported in 2020. This was attributed to COVID-19 outbreak.

**Table 1:** Characteristics of FGD participants

Characteristics	Frequency (***n*** = 292)	Percent (%)
**Sex**		
Female	167	57.2
Male	125	42.8
**Age group (in years)**		
20-29	32	11.0
30-39	101	34.6
40-49	94	32.2
50-59	45	15.4
60-69	20	6.8
**Role in health facility**		
Nurse	55	18.8
OIC/MD/Administrator	31	10.6
Lab Technician	28	9.6
Nurse Aide	26	8.9
IPC/Sanitation	21	7.2
Vaccinator	20	6.8
Registrar	19	6.5
Ward Supervisor	17	5.8
Surveillance Officers	17	5.8
Physician/Screener	16	5.5
Nursing Director	13	4.5
Dispenser	9	3.1
Midwife	8	2.7
Other	8	2.7
Security	4	1.4
**Employed after EVD**		
Yes	172	58.9
No	120	41.1
**Health facility location**		
Urban	30	73.2
Rural	11	26.8

**Table 2:** Themes, Sub-themes and some quotations identified through interview with healthcare workers

Themes	Sub-themes	Quotations
Disruption i.e., change or drop in hospital attendance	Service distruption	“… For instance, at the clinic prior to the COVID-19 outbreak, the monthly patient load was 400. But since the COVID-19 outbreak in March, the monthly patients load ranges from 30 to 50, people are afraid to come to hospital …. The monthly admitted male patients used to be 45-50. Since COVID-19, we admitted 11 per month. … .and only 80 women delivered in the health facility instead of the average 150 women per month.” - D 20: Private HF, “We were receiving 2000 clients for vaccination monthly but dropped to 900 -1000 since COVID-19.” - D 27: Private HF.
Causes of health service disruption	Patient’s fear of being diagnosed with COVID	“Because of fear, some sick patients preferred to die at home rather than come to the health facility and were diagnosed with COVID.”- D 28: Public HF. “… “Very ill patients refuse to be referred to the county’s referral hospital due to the fear of being isolated and suspected to have COVID-19 disease.”- D 34: Private HF.
	Prevent contracting infection by avoiding the health facility and health care workers	“… the closure of the health facility following a confirmed case of COVID-19 infection among health workers made the community members stigmatize health facility staff and refuse to seek health care in the health facility after reopening.” - D 3: Private HF, and D 33: Private HF
	Perception that healthcare workers will inject them and their children with COVID-19 vaccine	. “… health workers have been given money to infect our children with COVID-19 vaccine.” - D 13: Public HF. “Parents are no longer bringing their children for vaccination, … they think that people are moving around vaccinating children with COVID-19 vaccine.” D 34: Private HF.
Recovery strategy from health service disruption	Health promotion and awreness creation	“… the hospital set up a health promotion team that went into the camps to educate the community dwellers, especially for pregnant women and children vaccination.” - D 26: Private HF, “The communities are now cooperating with us; they are attending the hospital with the help of health education and awareness. Before they will not attend the facility if they have signs and symptoms of COVID-19.” - D 16: Public HF
	Reduce number of staff per shift	“… the health facility reduced the number of staff working per time on duty to prevent contacting COVID-19 infection.” - **D 3: Public HF**
	Close and reschedule clinic attendance for patients	. “… we postponed TB Clinic for a week for us to come up with a strategy of scheduling patients and observe social distancing.” -D 39: Public HF.

“During the COVID-19 outbreak, we were not having patients, our finances and everything went low. That was a great challenge to the facility because from the fund generated, we get our daily bread and purchase the medication we dispense for the patient that come.” **- D 40: Private HF.** “ … from the outbreak onset, we found it very difficult … At times, we will come here, and for the whole day, no patient will come. Fear and panic affected the district health team indicators in the HMIS report. We have absolutely zero indicators for vaccine coverage and some other services. So, it greatly affected us in our surveillance data collection and report.” **- D 35: Private HF.**

### Disruption in outpatient visits and inpatient admission

**Fig. 1:**
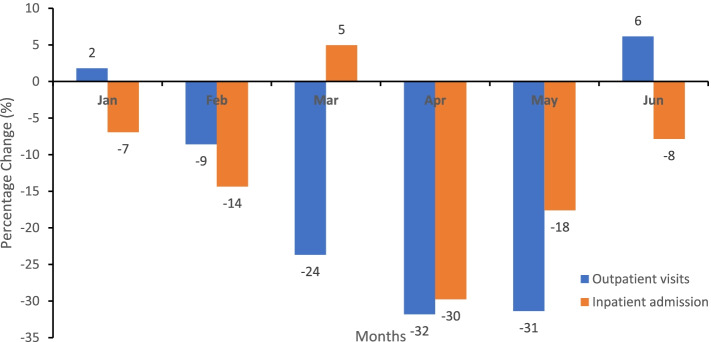
Disruptions or relative percentage change in the monthly outpatient visits and inpatient admissions, Liberia, Jan-Jun 2020

In 2020, from February to May, outpatient health facility attendance progressively decreased from − 9% in February 2020 to − 32% in April 2020 and inpatient admission from − 7% in January 2020 to − 30% in April 2020, as shown in Fig. 1. The lowest relative percentage change in outpatient visits and inpatient admission was in April 2020. The participants attributed the disruption in health facility attendance to the COVID-19 outbreak.

“… For instance, at the clinic prior to the COVID-19 outbreak, the monthly patient load was 400. But since the COVID-19 outbreak in March, the monthly patients load ranges from 30 to 50, people are afraid to come to hospital …. The monthly admitted male patients used to be 45-50. Since COVID-19, we admitted 11 per month. … .and only 80 women delivered in the health facility instead of the average 150 women per month.” **- D 20: Private HF**

“COVID-19 brought fear to patients. They refused sample collections for any test at the laboratory, and their attendance at the health facility was low.” **- D 13: Public HF.**

### Disruption in HIV/AIDs and malaria diagnosis and treatment services

**Fig. 2:**
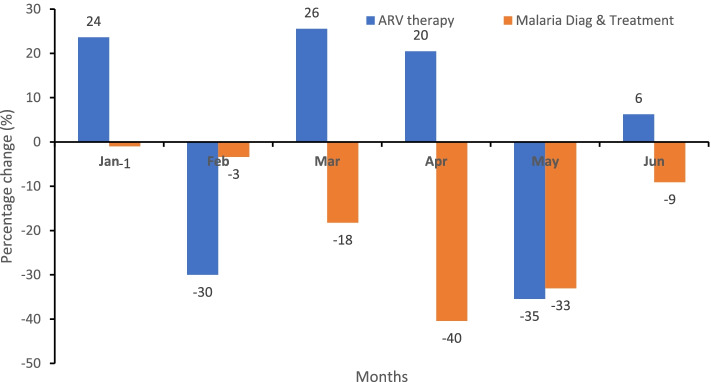
Percentage change in the level of HIV/AIDs and malaria services in 2020 compared to the same period in 2019, Liberia

For HIV/AID services, the relative percentage change in antiretroviral therapy was positive compared to 2019, but it decreased from 26% in March 2020 when the COVID-19 outbreak was declared to 20% in April 2020, sharply declined to − 35% in May 2020 and rebound to 6% in June of the same year, as shown in Fig. 2. Additionally, malaria diagnosis and treatment decreased progressively from a minus 3% change in patients receiving diagnosis and treatment for malaria in February 2020 compared to February 2019 to a minus 40% change in April 2020 and a minus 33% change in May 2020.

### Disruption in ante-natal care services

**Fig. 3:**
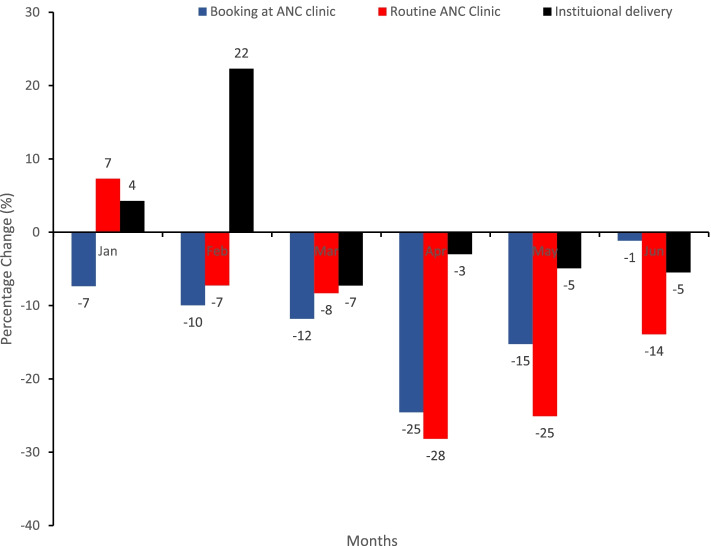
Relative percentage change in health facilities ANC services and institutional deliveries, Liberia, Jan – Jun 2020

Generally, in 2020 compared to 2019, there was a decrease in the relative percentage change in the number of pregnant women booking at antenatal clinics. However, the number of women receiving routine antenatal care decreased from − 7% in February 2020 to − 28% in April and − 25% in May 2020, whereas institutional delivery declined from 22% in February to − 7% in March and − 35% in April 2020 compared to the same period in 2019 (Fig. 3).

### Disruption in routine immunization services

**Fig. 4:**
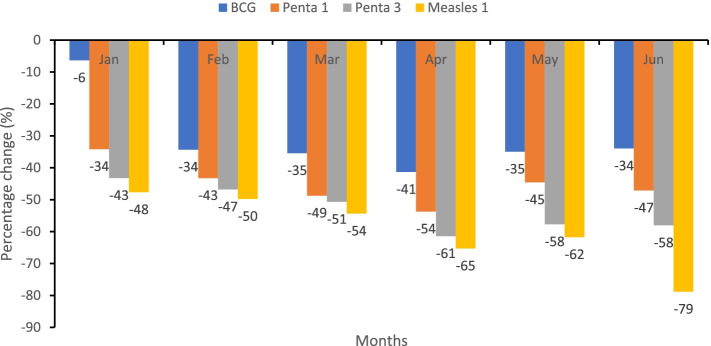
Relative percentage change decrease in the number of children receiving BCG, Penta 1, Penta 3, and measles vaccination, Liberia, Jan – Jun 2020

Routine immunization was offered by 92.7% (38/41) of the selected health facilities. Only 31.6% (12/38) conducted scheduled routine immunization outreaches in ≥80% of the communities targeted from January to June 2020. In Fig. 4, in 2020, the number of children vaccinated with BCG, Penta 1, Penta 3, and Measles decreased relative to 2019. Overall, the scheduled routine immunization outreaches that were conducted decreased by 47%, i.e., 290 outreaches were conducted from January to June 2020 compared to 547 in the same period in 2019. Generally, the attendance at the immunization clinic in 2020 was low.

“We were receiving 2,000 clients for vaccination monthly but dropped to 900 -1,000 since COVID-19.” - **D 27: Private HF**.

### Causes of disruption in routine healthcare services

Some of the reasons for the disruptions in routine health service from the participant’s perspective were the fear of either contacting COVID-19 infection or being diagnosed with COVID-19 and placed in isolation, inadequate PPE for healthcare workers use, unavailability of healthcare workers due to their redeployment to COVID-19 response, and due to the lack of ambulance referral services which diverted to support COVID-19 response, and none was available for referral of patients.

“Because of fear, some sick patients preferred to die at home rather than come to the health facility and were diagnosed with COVID**.”- D 28: Public HF.** “… patients were afraid and thinking that health professionals will impose positive COVID-19 results on them. One patient at the health facility said that “normally, before this COVID-19, we used to have ‘fresh cold’ and others, but now when we get a little sickness, you people (clinicians) will put us down and say that we have COVID-19. That is why we have been buying our medicine outside before you people come and grab us”. **- D 3: Private HF and D 34: Private HF.** “Very ill patients refuse to be referred to the county’s referral hospital due to the fear of being isolated and suspected to have COVID-19 disease.”**- D 34: Private HF.** “The health facility no longer has access to ambulance services because of its usage for COVID-19 response.” **- D 7: Public HF.**

However, other reasons for disruption were restriction of movement due to the lockdown, partial closure, or reduction in the routine healthcare services available in the health facility and no drugs in the health facility. Additionally, there was a stigma toward the healthcare workers working at the health facilities and reported COVID-19 infections among healthcare workers may account for the disruption in routine healthcare services.

“… the closure of the health facility following a confirmed case of COVID-19 infection among health workers made the community members stigmatize health facility staff and refuse to seek health care in the health facility after reopening.” **- D 3: Private HF, and D 33: Private HF**. “… community members were afraid of entering the hospital..., relationship with doctors who live in the community were restricted and community members distanced themselves from them.” **- D 3: Public HF.**

On the other hand, a lack of drugs and vaccines accounts for the disruption in routine healthcare services. Scheduled routine immunization outreaches were not implemented by the health facilities rendering immunization services due to the lack of logistic support in 29.0% (11/38), i.e., no motorbike, no gasoline, faulty refrigerator, etc., outright cancellation of immunization outreaches due to lockdown and restriction gathering in 18.4% (7/38), and stockout of vaccine in 5.3% (2/38). Vaccine stockout for more than 30 days between January and June 2020 was BCG 7.9% (3/38), Penta 13.2% (5/38), and measles 15.8% (6/38) in health facilities rendering immunization services.

The major reason for the disruption of immunization services was parental refusal to bring their children for vaccination due to fear of COVID-19 infection, as reported in 65.8% (25/38) of health facilities, and parents believed that their children would be injected with COVID-19.

“… parental misconceptions and fear caused the low utilization of health services like EPI services.” **- D 14: Private HF. “**… health workers have been given money to infect our children with COVID-19 vaccine.” **- D 13: Public HF.** “Parents are no longer bringing their children for vaccination, … they think that people are moving around vaccinating children with COVID-19 vaccine.” **D 34: Private HF.**

### Recovery strategies to mitigate routine healthcare service disruption in Liberia

Recovery strategies in health facilities to restore routine healthcare services to normal were diverse. The health facilities engaged community members through massive health education on COVID-19 prevention and control. They were informed of measures put in place in the health facility to protect everyone and improve routine health services, such as reduction in the number of healthcare workers per shift to maintain social distancing, rescheduling of patient appointments and strict compliance with the use of nonpharmacological preventive measures.

“… the hospital set up a health promotion team that went into the camps to educate the community dwellers, especially for pregnant women and children vaccination.” **- D 26: Private HF.** “… the health facility reduced the number of staff working per time on duty to prevent contacting COVID-19 infection.” - **D 3: Public HF.** “… we postponed TB Clinic for a week for us to come up with a strategy of scheduling patients and observe social distancing**.” -D 39: Public HF.**

Massive awareness was created in the health facility among the health facility’s catchment communities at the community meetings and religious and social gatherings to inform and persuade them of the safety of the health facility to render routine healthcare services.

“Initially, when the outbreak started, there was fear in the people that affected the utilization of the health care services until later when the proper awareness was created by giving the right information.” **- D 42: Public HF.**

“So, at that time, it was a problem for us, but the officer in charge of the health facility asked us to be talking to them, creating awareness that is how things start getting better even though presently we are not 100%.” **– D 40: Private HF.** “We were not even getting 50 persons daily, but with the continue health educations, patients load is rising gradually.” **- D 28: Public HF.** “The communities are now cooperating with us; they are attending the hospital with the help of health education and awareness. Before they will not attend the facility if they have signs and symptoms of COVID-19.” **- D 16: Public HF.**

## Discussion

This study used a mixed approach to describe the impact of the COVID-19 outbreak and disruption of routine healthcare services, reasons for the disruptions, and recovery strategies to revert routine healthcare services to normalcy in the health facilities at 6 months into the first epidemic wave of the COVID-19 outbreak in Liberia. As a novel disease, the COVID-19 pandemic evokes fear with significant diversion of resources and attention to curtail its transmission [15, 16]. Therefore, the delivery of routine health services in a weak and fragile health system in developing countries, including Liberia, was devastated by the pandemic [15, 16]. This study shows that the COVID-19 outbreak in Liberia disrupted routine healthcare services relative to January to June 2019; on average, there was an 18% decrease in malaria diagnosis and treatment [17], 15% in outpatient visits, 12% in inpatient admissions, 12% in antenatal services, and 1.2% in HIV/AID services. This study agreed with previous studies on the impact of the COVID-19 outbreak on the disruption of routine healthcare services [18–20] and was similar to the disruption caused by the Ebola virus disease (EVD) outbreak in Liberia and other neighboring West African countries [9]. However, there was a 0.3% increase in institutional delivery, which could be related to the declaration of the government to reduce maternal mortality, and there are penalties in the communities for any pregnant women with home delivery [21, 22].

Interventions to control the transmission of the COVID-19 pandemic have caused an indirect effect and widespread disruption in routine healthcare services such as immunization services [11] as well as malaria diagnosis and treatment [2, 15]. In Liberia, the government declared the COVID-19 outbreak on 16 March 2020 and instituted a total or partial lockdown on April 10 to July 12, 2020, similar to other countries, as one of the immediate control measures to stop COVID-19 transmission [23]. According to Dixit et al., 2021 [11], lockdown is defined as a highly restrictive set of nonpharmaceutical interventions against COVID-19, including either stay-at-home orders or interventions with an equivalent effect on movement in the population through restriction of movement [23]. The institution of lockdown measures to control the COVID-19 epidemic in Liberia temporarily caused a disruption in routine healthcare services, such as the implementation of scheduled routine immunization outreach services in the communities, reduced parental access to routine immunization services, and affected global supply chain networks, causing an inadequate supply of personal protective equipment (PPE), vaccines, drugs and diagnostics at the health facilities and disrupting health facility attendance, causing a reduction in fund generation to pay staff in private health facilities and providing essential services to run the health facility [2, 7, 11]. The inability to supply and distribute health products such as diagnostics and drugs directly affects the delivery of routine healthcare services [7]. Additionally, parental refusal to access immunization services for fear of contracting COVID-19 and redeployment of immunization staff to COVID-19 response were other reasons for the disruption of routine immunization services. The disruption of routine immunization services could erode the previous gain to control vaccine preventable diseases by lowering community herd immunity and predisposing patients to vaccine preventable disease outbreaks [12].

Apart from the restrictions due to lockdown, rumors, misinformation, poor perceptions of COVID-19, and stigmatization of healthcare workers were the other reasons for disruption in routine healthcare services [23, 24]. This study revealed that community members believed that healthcare workers would inject them with the COVID-19 vaccine or impose a COVID-19 diagnosis on them and place them in isolation if they visited health facilities to access routine healthcare services. Therefore, they prefer to either visit patent medicine vendors to purchase medication or die at home rather than utilizing routine healthcare services at the health facility. Additionally, COVID-19 infections among healthcare workers working in health facilities caused stigma towards the healthcare worker and indirectly disrupted access to routine healthcare services by the community. The study agreed with other studies [2, 7, 25] from Europe and America that identified cancellation and rescheduling of scheduled hospital appointments, fear of contacting COVID-19 infections while visiting health facilities, and shortages of healthcare workers due to their redeployment to support the COVID-19 outbreak response as causes of disruptions in routine healthcare services [11].

The World Health Organization and partners advise countries to implement recovery strategies to mitigate disruptions in routine healthcare services [2, 26]. Liberia used other measures, unlike other countries that used telemedicine [27] to avert disruption in routine healthcare service delivery during the COVID-19 pandemic. Following the disruptions in routine health service delivery and the directives from the WHO, the government issued a directive to the health facilities to recommend and sustain routine healthcare service delivery. Therefore, health facilities commenced massive strategic health education and communications [11] with community members on the use of COVID-19 preventive and control measures. The health facilities also modified the health facilities service procedures to provide a safe environment for patients to assess routine healthcare services. Health education aimed to demystify myths, misinformation, and misperceptions of COVID-19 [2] among the communities in the catchment area.

Although the COVID-19 outbreak was declared on March 16, 2020, the disruption of routine healthcare services peaked from April and May 2020. The institution of strategic recovery measures by health facilities yielded a quick reversal of the disruption of routine healthcare services by June 2020.

## Conclusions

The COVID-19 outbreak in Liberia resulted in the disruption in routine healthcare service delivery during the first epidemic wave. The disruption affected all routine healthcare services assessed by hindering the supply and demand of routine healthcare services. The prompt and effective implementation of diverse recovery strategies by health facilities directed the recovery pathway and restoration of routine healthcare services to the pre-COVID-19 era.

### Limitation

This study is not without limitation. Administrative data were used for the quantitative assessment of healthcare service disruptions. Therefore, they are subjected to the flaws associated with secondary data. However, efforts were made to obtain quality data collected used in the analysis. Additionally, the study was a rapid survey conducted to respond to the disruption of routine healthcare services. The health facilities selected were purposeful and secondary-level health facilities, which may not adequately represent the real situations at primary-level health facilities that are not preselected as COVID-19 precautionary observation centers and tertiary health facilities. Additionally, attempts to involve all levels of the health system are preferred for generalization of study outcomes.

### Recommendations

To avoid healthcare service disruption during the pandemic, the Ministry of Health should implement strategies to avoid redirecting all healthcare resources to the outbreak or epidemic response and sustain the delivery of routine healthcare services. Depleting the distribution of insufficient health workforce by transferring them to support COVID-19 outbreak response without providing for surge health workforce should be avoided. The adoption and implementation of strategies to curb COVID-19 transmission should be well considered during the preparedness and response plan vis-à-vis strategies to sustain routine healthcare service delivery during the outbreak. Such strategies like provision of infection prevention and control materials for healthcare workers use, enhance public awareness and avoiding movement restriction will indirectly prevent the disruption of the routine care services.

## Data Availability

The datasets generated and/or analyzed during the current study are not publicly available due to their containing information that could compromise the privacy of research participants but are available from the corresponding author on reasonable request with approval from national COVID-19 incident management.

## Abbreviations

EPI: Expanded Program on Immunization
EVD: Ebola Virus Disease
HFSFP: Health Facility Surveillance Focal Person
IPC: Infection Prevention and Control
OIC: Officer-in-Charge
PHEIC: Public Health Emergency of International Concern
PPE: Personal Protection Equipment
SOP: Standard Operation Procedure
UNICEF: United Nations International Children’s Emergency Fund
WHO: World Health Organization
